# Effect of Diet and Essential Oils on the Fatty Acid Composition, Oxidative Stability and Microbiological Profile of Marchigiana Burgers

**DOI:** 10.3390/antiox11050827

**Published:** 2022-04-24

**Authors:** Isa Fusaro, Damiano Cavallini, Melania Giammarco, Annalisa Serio, Ludovica M. E. Mammi, Julio De Matos Vettori, Lydia Lanzoni, Andrea Formigoni, Giorgio Vignola

**Affiliations:** 1 Faculty of Veterinary Medicine, University of Teramo, Località Piano D’Accio, 64100 Teramo, Italy; ifusaro@unite.it (I.F.); jdematosvettori@unite.it (J.D.M.V.); llanzoni@unite.it (L.L.); gvignola@unite.it (G.V.); 2 Department of Veterinary Medical Sciences, Alma Mater Studiorum University of Bologna, Via Tolara di Sopra 50, Ozzano dell’Emilia, 40064 Bologna, Italy; damiano.cavallini@unibo.it (D.C.); andrea.formigoni@unibo.it (A.F.); 3 Faculty of Bioscience and Technology for Food, Agriculture and Environment, University of Teramo, Campus Coste Sant’Agostino, Via R. Balzarini 1, 64100 Teramo, Italy; aserio@unite.it

**Keywords:** TBARS, FRAP, colour parameter, linseed and vitamin E, oregano and rosemary, microbiological profile, beef meat

## Abstract

The objective of this study is to evaluate the effects of including linseed (L) or linseed plus vitamin E (LE) in the diet of Marchigiana young bulls on the oxidative stability, color measurements, microbiological profile and fatty acid composition (FA) of burgers treated with and without a blend of essential oils (*Rosmarinus officinalis and Origanum vulgare var. hirtum*) (EOs). For this aim, the burgers were analysed for pH, thiobarbituric-acid-reactive substance (TBARS) content, Ferric Reducing/Antioxidant Power Assay (FRAP), vitamin E and colour measurements (L, a*, b) at 3, 6, 9, 12 days of storage: the TBARs were the highest in group L compared to C and LE after 12 days of storage (0.98, 0.73, and 0.63 mg MDA/kg, respectively). The TBARS content was also influenced by the use of EO compared to burgers not treated with EO (*p* < 0.05). The vitamin E content was influenced by the diet (*p* < 0.01), but not by the EO. The meat of the L group showed the lowest value of redness (a*) compared to C and LE (*p* < 0.01), while the use of EO did not affect colour parameters. The microbiological profile of the burgers showed a lower Pseudomonas count for L and LE at T0 (2.82 ± 0.30 and 2.30 ± 0.52 Log CFU/g, respectively) compared to C (3.90 ± 0.38 Log CFU/g), while the EO did not influence the microbiological profile. The FA composition was analysed at 0 and 12 days. The burgers from the LE group showed the highest value of polyunsaturated FA compared to the L and C groups (*p* < 0.05). Our findings suggest that the inclusion of vitamin E in a concentrate rich in polyunsaturated fatty acids is useful to limit intramuscular fat oxidation and to preserve the colour stability of burgers from young Marchigiana bulls enriched with healthy fatty acids. Moreover, linseed and vitamin E had a positive effect on microbial loads and growth dynamics, containing microbial development through time.

## 1. Introduction

In Italy, as in other European countries, the purchase of processed foods, such as meat burgers and patties, has tripled in the last 50 years probably due to changes in the Italian lifestyle, preferring food that is faster to cook [1]. This is also probably due to their price and preparation versatility as well as changes in eating habits, the availability of a different kind of meat product and sociodemographic changes [2]. Nonetheless, the meat from ruminants is considered a major source of saturated fatty acids (SFAs) because red meat consists of approximately 40% of SFAs, 50% monounsaturated fatty acids (MUFAs), 5% trans fatty acids and 4% polyunsaturated fatty acids (PUFAs) [3]. The main daily meals in Western countries include a meat-containing dish that contributes to approximately half of the maximal recommended intake of SFAs [4,5].

Several large observational studies have reported strict associations between SFA concentrations in the diet and several health problems, ranging from cardiovascular disease (CVD) to cancer risk [6,7]. Conversely, there is emerging evidence that a diet rich in PUFAs, in particular with three double bonds like n-3 fatty acids (n-3FAs) acids and conjugated linoleic acids (CLAs), has beneficial effects on human health, such as decreasing low-density lipoprotein and cholesterol, and conferring anti-inflammatory, anti-atherogenic and anti-carcinogenic effects [8]. The feeding strategies were demonstrated to be the best approach to reduce SFAs in meat and, in general, in ruminants’ products [9]. It is well known that the use of pasture or the introduction of vegetable oils in the diet of ruminants can induce the switch in meat and milk fatty acids (FAs) from saturated to unsaturated, as demonstrated in lamb [10], ewe [11], dairy cattle [12] and beef [13]. The use of feed rich in polyunsaturated fatty acids represents an innovation and enhancement in the breeding methods of typical local breeds such as Marchigiana, Chianina and Romagnola, in Italy. The husbandry techniques for the Marchigiana breed plan a diet rich in cereal-based feed during the finishing period [14]. For this reason, it could be interesting to add feed that can enrich beef meat with PUFAs. However, adding PUFAs to the meat makes lipid oxidation easier, with abrupt consequences on meat sensorial and chemical characteristics [15]. In particular, the meat’s color is a fundamental sensory attribute that influences the consumers’ choice of purchasing it, and, for this reason, it is necessary to maintain its attractive nature for the entire shelf-life period [16]. In this context, the use of natural antioxidant spices may be useful to contrast the increased oxidation due to the high concentration of unsaturated fatty acids, especially in the ground meat products. For example, the application of essential oils (EOs) has been demonstrated as helpful to protect meat products against oxidation [16]. Essential oils are extracts obtained by the distillation of a wide variety of plant materials, and they exert both antioxidant and antimicrobial effects, depending on their chemical composition. The interest of researchers and industries in their exploitation has recently increased, as EOs are considered natural compounds, capable of substituting synthetic preservatives, and are thus particularly appreciated by the consumers [17]. 

In the light of these considerations, the aim of this research is to evaluate the effects of a concentrate enriched with linseed and vitamin E on oxidative stability colour measurements, microbiological profile and fatty acids composition of meat burgers from young Marchigiana bulls. Moreover, aiming to improve the quality attributes of the burgers, *Rosmarinus officinalis* and *Origanum vulgare* var. *hirtum* essential oils are included in the burger formulations and their effects are evaluated during the meat storage.

## 2. Materials and Methods

### 2.1. Animals and Diets

The Marchigiana young bulls were managed during the experiment according to the Council Directive 98/58/EC of 20 July 1998 concerning the protection of animals kept for farming purposes, and were slaughtered according to the Council Regulation (EC) No 1099/2009 of 24 September 2009 on the protection of animals at the time of slaughtering. The experiment was conducted, as previously described by Fusaro et al., 2021 [18]. Briefly, a total of 36 Marchigiana young bulls bred in a commercial farm approximately 15 km northeast of Teramo, Abruzzo, Southern Italy were allotted in three groups receiving three different experimental diets (Table 1): Control (C), Linseed (L) and Linseed plus Vitamin E (LE). The groups were homogeneous for body weights (441.9; 438.8 and 440.1, for C, L and LE, respectively). Samples of Total Mixed Ration (TMR) were collected every week and analysed according to the standard methods of AOAC (2002) [19] for dry matter (DM), crude protein (CP), ether extract (EE) and ash. Neutral detergent fibre (NDF), acid detergent fibre (ADF) and acid detergent lignin (ADL) were determined, as previously described [19,20]. All feed samples were also analysed for FA composition, as described in the research [21]. The ingredients and chemical compositions of the diets are shown in Table 1.

### 2.2. Preparation of the Burgers 

The meat from the left of the *Longissimus dorsi* muscle between the last rib and the 6th lumbar vertebra of each carcass was ground in a meat grinder with a 3 mm disk. From each carcass, 8 burgers 25 mm thick were obtained. Four burgers were kneaded with a blend of *Rosmarinus officinalis* and *Origanum vulgare* var. *hirtum* (1:1) EO (0.05 mL rosemary + 0.05 mL oregano per kg of meat) for 60 s, and the remaining burgers were kneaded with an equal amount of PBS (Phosphate Buffer Saline) as a control. PBS is isotonic and non-toxic. All samples (*n* = 288) underwent modified atmosphere packaging (MAP) (66% O_2_; 25% CO_2_; 9% N_2_) and were then stored at +4 °C for 12 days and sampled for the subsequent analysis. Whole trays were placed in a dark chamber at 4 °C and then removed from the chamber for the analysis. 

### 2.3. Meat Quality: Chemical Analyses, pH and Colour Measurements

At time 0, all the burgers were analysed for moisture, fat, protein and ash [19]. The pH, TBARs, FRAP, Vitamin E and colour parameter were analysed at 0, 3, 6, 9 and 12 days of storage [22]. The pH of the meat samples was measured with a penetrating electrode adapted to a portable pH meter (Crison pHmeter 507 and a 52–32 spear electrode, Crison Instruments, Spain). Burger samples from each treatment were subjected to pH recording during storage (0, 3, 6, 9 and 12 days of storage). At the same time points, meat colour parameters of lightness (L) redness (a*) and yellowness (b) were measured for the burgers, according to the CIELab system, with a Minolta Chroma Meter CR-300 (Minolta Camera Co., Osaka, Japan) with a D65 illuminant and an 8 mm aperture. The burgers were allowed to bloom in direct contact with air for 1 h before colorimetric measurements were performed on the burger surfaces and reported as the mean of three measurements. 

### 2.4. Burgers Oxidative Stability: Thiobarbituric-Acid-Reactive Substances (TBARS), Ferric Reducing Antioxidant Power (FRAP) and Vitamin E

The lipid oxidation of meat samples was evaluated by TBARS measurement with the method of Inserra et al., (2014) [23] at 0, 3, 6, 9 and 12 days of storage. Three replicates were run for each sample. At the same time points, the spectrophotometric FRAP method was used for the antioxidant capacity determination according to previous studies [24]. Muscle vitamin E (α-tocopherol) concentration was measured at the same time points according to Koprivnjak et al. (1996) [25].

### 2.5. Fatty Acid Analysis

The fatty acid analysis was conducted on burgers from the three experimental groups at time point 0 and 12. Briefly, intramuscular lipids were extracted according to the protocol of Folch et al. (1957) [26]. After cold methylation with the technique of Frega and Lerker (1984) [27], the FAs were determined by gas chromatography with a Chrompack CP-SIL 88 capillary column. Before statistical analysis, the data on FA composition were processed to calculate the following FA classes: MUFA, PUFA and SFA; n-3 (Σ n-3: sum of C18:3n-3, C20:5n-3, C22:5n-3 and C22:6n-3); and n-6 (Σn-6: sum of C18:2n-6t9,t12, C18:2n-6, CLAt10,c12, C18:3n-6, C20:3n-6, C20:4n-6 and C22:4n-6) [28]. The I-Harris index [29] was calculated as the sum of EPA and DHA. FA quantities were expressed as mg FA/100 g sample.

### 2.6. Microbiological Analysis

To evaluate the microbial population during the burgers’ shelf-life, 10 g of meat from each burger were homogenised with a sterile saline solution (NaCl 9 g/L in deionised water) in a ratio of 1:10 (*w*/*v*) for 300 s in stomacher. Afterwards, ten-fold serial dilutions were prepared, and the following microbial groups were determined as follows: Mesophilic and Psychrotrophic Aerobic Counts were determined on Plate Count Agar at 30 °C for 48 h and at 8 °C for 7 d, respectively; presumptive lactobacilli in MRS at 30 °C for 48 h; presumptive lactococci on M17 agar at 30 °C for 48 h; coliforms in violet red bile agar (VRBA) at 37 °C for 48 h; staphylococci on Mannitol Salt Agar at 37 °C for 48 h; *Pseudomonas* spp. on Pseudomonas Isolation Agar (Acumedia, Dot Scientific, Burton, MI, USA) at 22 °C for 48 h and *Brochothrix thermosphacta* on STAA agar base added with STAA selective supplement at 25 °C for 48 h. Where not differently specified, all the culture media were from Oxoid Thermo Fisher (Rodano, Italy). The samples were analysed in triplicate and the mean of the results was calculated.

### 2.7. Statistical Analysis

All data were analysed with a GLM procedure using JMPpro v16.0 (SAS Institute Inc., Cary, NC, USA). Meat quality composition at time point zero was analysed using diet as the only fixed effect. Data relative to oxidative stability, colour measurements, microbiological profile and fatty acid composition were analysed, including the fixed effects of diet, EO and time of storage and their interactions.

Results for interactions between the main effects are shown in Tables and Figures. Tukey’s test was performed to assess significant differences between means. *p*-value ≤ 0.05 was considered the threshold for significant differences. Results are presented as treatment mean and standard error of the mean.

## 3. Results

### 3.1. Beef Burger Characteristics: Chemical Analyses, pH, Color Measurements and Oxidative Stability

The chemical composition of the burgers is presented in Table 2. The moisture contents ranged from 74.79 to 75.45% showing no differences among the meat samples from three groups. The different diets did not influence the lipid, protein and ash content of the meat (*p* > 0.05).

Table 3 presents the pH, oxidative stability (TBARS, FRAP and vitamin E) and colour parameters (L, a* and b) of the burgers from the three experimental groups, treated with or without EO and their interactions. The experimental diets significantly affected the burgers’ pH (*p* < 0.05), showing a lower value in the meat of group L (5.60) compared to group LE (5.67), while a higher value was registered for group C (5.70). The burges’ pH was also affected by the use of EOs (*p* < 0.03), showing a higher value for the samples treated with EOs compared to the meat without EOs (5.47 vs. 5.65, respectively). The TBARS content of the burgers showed a higher value in group L (0.60 MDA/kg of meat) compared to the C and LE groups (0.47 and 0.39 MDA/kg of meat) (*p* < 0.05). Moreover, the use of EOs influenced the TBARS value (*p* < 0.05), but not the vitamin E content (*p* > 0.05). Vitamin E content was higher in the LE group compared to C, while the L group showed an intermediate value (*p* < 0.05). The FRAP showed differences among the dietary treatment and between the burgers treated and not treated with EOs. The burgers of the LE group had a higher FRAP (0.74 μmolFe/g) compared to those of the C and L groups (0.54 and 0.55 μmol Fe/g, respectively, in C and L). The FRAP was also influenced by the use of EOs (*p* < 0.05), showing the highest value in the samples treated with EOs (0.67 μmolFe/g) compared to the burgers without EOs (0.52 μmolFe/g).

The lightness was higher (*p* < 0.01) in the L group compared to the LE and C groups. Conversely, the redness was lower in the L group and higher in the LE and C groups (*p* < 0.01). The yellowness was lower in the L group compared to the C and LE groups (*p* < 0.01). The use of essential oils did not influence the colour parameter (*p* > 0.05)

**Figure 1:**
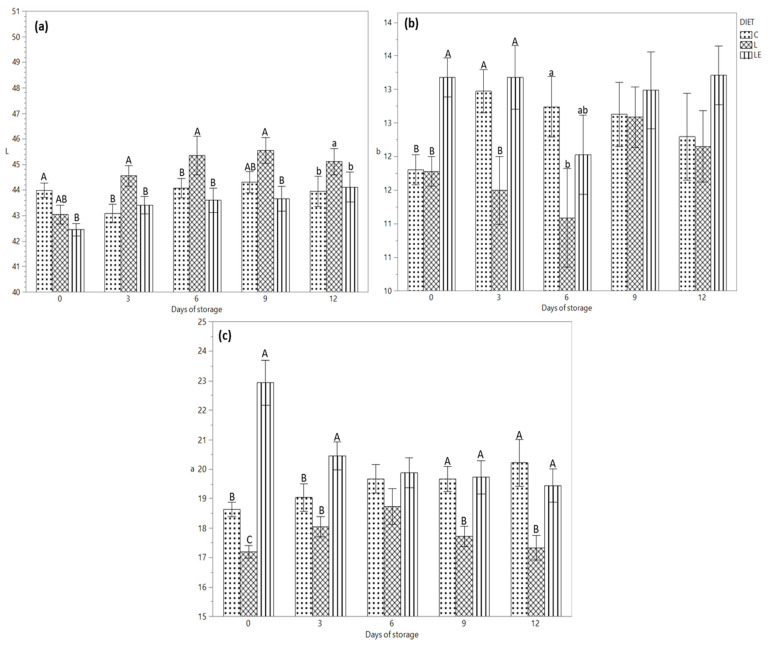
(**a**) Lightness, (**b**) Yellowness and (**c**) Redness of burgers from Marchigiana beef from three different dietary treatments, according to the storage time. Different letters indicate significant differences (a, b: *p* < 0.05; A, B, C: *p* < 0.01) within each time point. C = Control; L = Linseed; LE = Linseed plus vitamin E.

Figure 1a–c shows the differences between the colour parameters during the storage time. Figure 1a shows that the lightness value was higher from day 3 to day 12 of storage in group L compared to that of group LE, while the C group had an intermediate value on days 0 and 9 of storage (*p* < 0.01). Moreover, the interaction between time of storage and diet was significantly correlated (*p* < 0.01). The yellowness of the burgers (Figure 1b) was affected by the diet (*p* < 0.01), showing a higher value in the LE group compared to the C and L groups on day 0; on day 3 (*p* < 0.01), the b value was similar between the C and LE groups and lower for the L group, while on day 6, the yellowness of the meat of the L was similar with that of the LE and C groups, even if C and LE showed different yellowness value (*p* < 0.05). The redness shown in Figure 1c has a higher value for group LE (storage time point “0”) compared to groups C and L (*p* < 0.01). On day 3 of storage, the C and L groups showed a similar value compared to the LE group (*p* < 0.01), while on days 9 and 12 of storage, the redness was lower in the L group compared to the C and LE groups (*p* < 0.01). The interaction between the storage time and experimental diets significantly affected the redness of the meat (*p* < 0.01).

**Figure 2:**
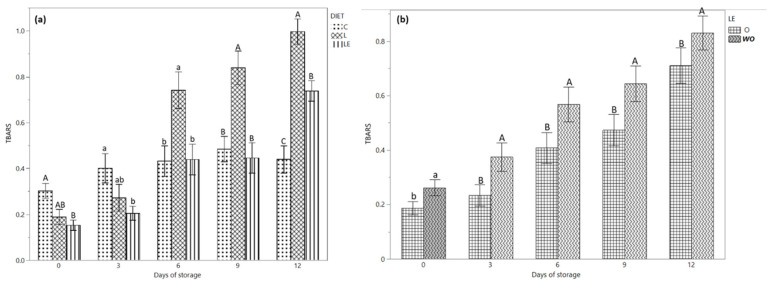
(**a**) TBARS values of the burgers from Marchigiana beef from three different dietary treatments according to the storage time. **(b)** TBARS values of the burgers treated with and without EOs according to the storage time. Different letters indicate significant differences (a, b: *p* < 0.05; A, B, C: *p* < 0.01) within each time point. C = Control; L = Linseed; LE = Linseed plus vitamin E. O = with essential oil; WO = without essential oil.

Figure 2a shows the TBARS content during the storage time according to the three different dietary treatments. On days 0 and 3 of storage, a higher concentration of TBARS content was observed in group C compared to group LE (*p* < 0.05). Conversely, on day 6 (*p* < 0.05) and 9 (*p* < 0.01) of storage, the TBARS of group L was higher compared to that of groups C and LE; on day 12, the C group showed the lowest value compared to group LE, while group L had the highest value (0.73, 0.98 and 0.63 mg MDA/kg in group C, L and LE, respectively).

Figure 2b and Table 3 show the TBARS content according to the storage time of burgers treated with and without essential oils. At all the time points, the use of EOs was correlated with a lower concentration of TBARS compared to the samples not treated with EOs.

### 3.2. Microbiological Profile

**Figure 3:**
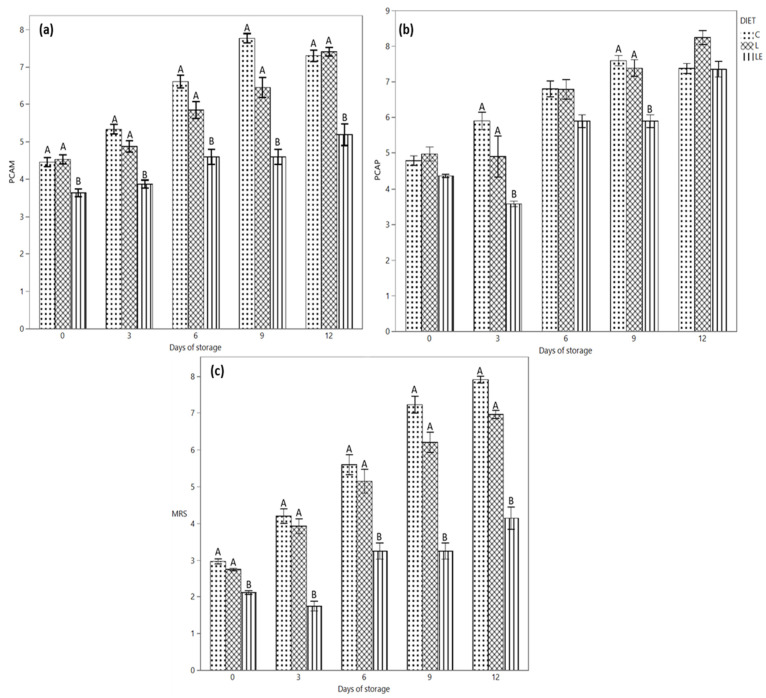
(**a**) Evolution of the mesophilic aerobic population, (**b**) psychrotrophic aerobic population and (**c**) lactic acid bacteria during the refrigerated storage of Marchigiana beef burgers in a modified atmosphere packaging. C = Control; L = Linseed; LE = Linseed plus vitamin E. Different letters indicate significant differences (A, B: *p* < 0.01) within each time point.

The microbiological profile of the samples is depicted in Figure 3. Regarding the microbiological profile of the products, a difference in mesophilic (Figure 3a) and psichrotrophic (Figure 3c) counts was revealed for all the samples at all the time points of the analysis. In particular, LE samples showed lower microbial counts in comparison with the other groups. The difference was already clear on day 0, until 12 days of refrigerated storage, when the mesophilic count only slightly increased for LE samples; it rose sharply for the L and C samples without and with essential oils. Smaller differences among the samples were observed for the psychrotrophic count; nevertheless, additionally in this case, LE samples showed the smallest counts, particularly in the first six days of storage. As regards the specific microbial groups analysed, the count trend during time reflected the previous behaviour, with the LE samples showing the lowest counts, particularly for presumptive lactobacilli (Figure 3b) and lactococci and then also for *Bhrochotrix thermosphacta* and *Pseudomonas* spp. (data not shown). In particular, the lower *Pseudomonas* counts observed already at T0 for the L and LE groups (2.82 ± 0.30 and 2.30 ± 0.52 Log CFU/g) with respect to group C (3.90 ± 0.38 Log CFU/g) allowed the control of this microbial group below the 5.00 Log CFU/g acceptable counts until the end of storage. On the contrary, microbial loads greater than 6.00 Log CFU/g were observed for the control samples starting from 9 days of storage.

For each sampling time, coliforms and staphylococci were below the detection limit for samples L and LE.

Regarding EO application, not significant (*p* > 0.05) differences were observed for EO-treated and -untreated samples within the same kind of samples in the first 3–6 days of storage, while differences increased during longer storage times, with different behaviour depending on the animal diet and the target microbial groups, which could present counts even higher in the EO-treated samples (data not shown). In any case, the differences were not statistically significant.

### 3.3. Fatty Acid Profile

The FA composition of burgers is shown in Table 4. Dietary linseed supplementation significantly increased the content of PUFA to 253.63 and 226.48 mg/100 g in the LE and L samples, while the lowest value was recorded for the C group (203.22 mg/100 g of sample). Moreover, the lowest value for MUFA was 595.53 mg/100 g in the sample in the C group, while the LE group sample had the highest value (699.22 mg/100 g of sample) and the intermediate vale was observed for the L group (595.53 mg/100 g of sample). SFA had an opposite trend, showing the highest value for group C (956.99 mg/100 g of sample) compared to groups L (721.53 mg/100 g of sample) and LE (679.51 mg/100 g of sample). Groups L and LE also showed a higher content of n-3 PUFA than group C, although these FA were higher in group LE (68.40 mg/100 g of sample) than in group L (49.43 mg/100 g of sample; *p* < 0.05). The total n-6 PUFA value was not affected by the diet (*p* = 0.68). The burgers from groups L and LE, compared with group C, had higher percentages of EPA and DHA (I-Harris index; 8.21 and 8.85 mg/100 g sample in L and LE, respectively, and 7.25 mg/100 g of sample in C; *p* < 0.05). In contrast, the CLA value was higher in the LE group (15.60 mg/100 g of meat) and lower in the C group (5.79 mg/100 g of sample; *p* < 0.05), whereas the L group had an intermediate value (11.22 mg/100 g sample). The saturation index was different among the dietary treatments, showing a higher value for the C group (1.02 mg/100 g sample) than L and LE groups (0.7 mg/100 g sample). The use of essential oils had no difference in the treatments.

## 4. Discussion

The experimental diets did not affect the meat’s chemical composition, showing similar results in each experimental group regarding protein, fat and ash content (Table 2). Generally, the meat composition is strictly related to the diet composition and, as described by other authors [30,31], the similar energetic and protein content of the experimental diets used in this study did not reveal differences in the protein, fat and ash content of meat.

Table 3 shows the effects of diets (C, L and LE) and the use of essential oils (O and WO) on Marchigiana beef burgers’ oxidative stability, pH, colour measurements and vitamin E content. In accordance with Juárez et al. (2012) [32], our results show that vitamin E had a role on the oxidative status of meat in the LE group, which showed a decrease in TBARS and an increase in FRAP values compared to the burgers from beef on diets without vitamin E. Additionally, the use of EOs influenced the oxidative parameters, demonstrating the ability of EOs to prevent oxidation [33,34].

In the present study, the oxidative stability and colour measurements of the burgers were significantly influenced by the three different experimental diets, showing that the burgers from the LE group were characterised by a lower TBARS content and a higher redness during storage. This effect could be due to the use of vitamin E during the finishing period, which seems to be considered an appropriate pre-slaughter feed strategy. In contrast to our study, a recent work on Normand cull cows showed that a diet supplemented with vitamin E (155 IU/kg of diet DM) and plant extracts rich in polyphenols did not prevent the lipid oxidation of the meat, with an increase in MDA concentration [35]. This discrepancy could be probably due to the greater amount of vitamin E in the diet of the LE group in the present experimental trial than in previous studies [35,36]. Indeed, as we expected, the concentration of vitamin E was higher in group LE compared to the other two groups, showing that the use of vitamin E in the diet influences their concentration in the meat. A large number of studies have observed the antioxidant effects of vitamin E to prevent the oxidation [36,37].

Figure 1a shows that the lightness was influenced (*p* < 0.01) by the diet and time of storage. In particular, the higher value of lightness was recorded in the burgers of the L group from day 3 to day 12 of storage compared to the LE and C groups. This result agrees with that reported by Juárez et al. (2012) [32], which found the lightness in patties increased when flaxseed without vitamin E was included in the diet. On the contrary, a greater pigment content in the diet implies a stronger light absorption and consequently lower reflectance or transmittance in the meat, making the product opaquer. Moreover, the colour parameters could be also influenced by the increase in oxidation level (TBARS), which in the burgers of group L was higher, due to an abrupt reduction in mitochondrial respiratory activity, which determines a great production of metmyoglobin [38]. A recent review on the effect of bioactive dietary nutrients on meat oxidation and colour stability has shown that in most ruminant studies the greatest colour stability and the least peroxidation were obtained in the meat of animals fed a super-nutritional dose of vitamin E. [39]. Our results also demonstrated for the first time that the oxidative stability and colour measurements of burgers from young Marchigiana bulls could be preserved when a high dosage of vitamin E is added during the finishing period. Some authors argued that the lightness could be partially influenced by differences in the intramuscular fat content [40]. In our study, we supposed that the difference in lightness could be due to the FA composition of the burgers because the content of intramuscular fat was similar between the diets (Table 3), as also demonstrated in dairy goat kids [41]. Moreover, in the current study, the higher concentration of vitamin E in the meat of the LE group increases stability against oxidation and makes the meat more desirable [32]. In contrast, Fusaro et al. (2021) [18] have reported that a dietary regimen with linseed and vitamin E was strictly related to a higher L value in steak from Marchigiana beef. Probably, in the ground beef as the one that was used in this study, the cellular integrity is likely to be disrupted, owing to the greater exposure of tissues to oxygen and the simultaneous dilution of antioxidant concentration.

Both storage time and experimental diets also affected (*p* < 0.05) the yellowness. In the control and LE burgers, the b value was higher on days 3 and 6 of storage. Conversely, in group L, the b fluctuated during the storage time with a lower value compared to that of the C and LE groups, probably due to auto-oxidation to brown metamyoglobin (Figure 1b), induced by higher lipid oxidation. This result was not observed in the LE samples, which is consistent with the antioxidant role of vitamin E.

The redness decreased from day 0 to day 12 in the burgers from all the three experimental groups. A decrease was registered in the L group at the end of the storage time (Figure 1c), which is probably due to the easier oxidation of red oxymyoglobin to brownish metmyoglobin determined by the presence of reducing systems and on lipid oxidation, as also demonstrated in burgers from lamb meat [42]. Moreover, the a* value of the burgers at time point 0 was significantly higher in group LE compared to the other two groups, probably due the antioxidant effect of vitamin E that could have protected the heme pigments of meat from oxidation [43].

The TBARS test was used to predict the oxidative stability of lipids in burger samples. It was demonstrated that the rancid smell and taste became detectable by the consumers when a value of 2 mg/kg of TBARS is found in the meat or meat products [32]. The TBARS values were significantly influenced by the diet, storage time and the use of EOs (Table 2 and Figure 2a,b). Our results show a higher degree of oxidation in burgers from the meat of animals fed with linseed, a lower degree in the control group and animals that received linseed and vitamin E. The time of storage also influenced (*p* < 0.01) the concentration of TBARS in all the burgers from the three dietary treatments, even if on days 6 and 9 of storage, the concentration of TBARS was constant in the LE and C groups, while higher in the samples from the L group. Conversely, during the first three days of storage, the group LE showed a lower level of TBARS compared to the L and C groups, while after 12 days, the TBARS had an intermediate value in groups L and C. Our results show a significant increase in TBARS in the burgers of animals that received a diet rich in linseed without the addition of vitamin E as also demonstrate by Juarez et al. (2012) [32], who found that beef from animals fed only with flax seeds showed a higher oxidation level compared to the animals that received a supplementation of vitamin E. The antioxidant capacity of vitamin E was evident in the burgers of the LE group compared to group L [44]; the results from the current research suggest that the oxidative stability of burgers from Marchigiana beef may be directly influenced by the use of antioxidants compounds, such as vitamin E and EOs, more than the fatty acid profile of intramuscular fat. To the best of our knowledge, this is the first study in which minced meat was used without adding fat in burger preparation. Indeed, as reported by Wang et al. [45], the beef patties experienced a greater rate of lipid oxidation and discoloration when a greater amount of fat is used for their preparation.

Our results also suggest that the use of EOs may be a favourable strategy to limit the oxidation process in meat burgers. This result could be attributed to the high concentration of phenolic compounds in EOs as well as other substances, such as flavonoids, which are responsible for antioxidant activity [46]. The food industry widely utilises a rosemary extract for its antioxidant properties [47]. The efficacy of rosemary in decreasing lipid oxidation has been reported by several authors in meat from poultry, pork [48] and beef [49]. Oregano extracts have also been demonstrated to be effective in inhibiting peroxidation because it blocks free radical formation [50].

In our experiments, burgers from control animals and those fed with linseed showed similar microbial loads and growth dynamics. On the contrary, LE samples generally had a better microbiological profile with lower counts. Therefore, our data demonstrate that the animal’s diet could significantly affect both the microbiological profile of the meat and its evolution during storage, having a direct impact on the improvement of the whole quality profile.

As regards the details, the most relevant data are associated with the mesophilic population as well as lactic acid bacteria (LAB). According to the results, the MAP packaging selected LAB and *Brochothrix thermosphacta*, which are usually the dominant spoiling microorganisms in the applied conditions [16]. As a consequence, the significantly lower counts (*p* < 0.05) observed in the LE samples are an important result to delay the spoilage. As the gas mixture applied contained a high percentage of oxygen, *Pseudomonas* spp. were also able to grow during burger storage, again with lower counts in the LE and L samples. *Brochothrix thermosphacta* and *Pseudomonas* spp. greatly contribute to the development of off-odours and off-flavours during meat storage, as the first microorganism produces cheesy and acid odours, while the latter is responsible for sulphuric, putrid, sweet and fruity odours [51]. The different diet supplementation of the animals seems to have a significant effect on the microbial population of the derived burgers, in particular in terms of microbial load. In fact, the supplementation with linseed and vitamin E improves the microbiological quality of the products, with lower counts not only at time 0, but also during the whole storage at 4 °C. Depending on the bacterial group considered, the meat from animals fed with linseed without vitamin E also shows lower counts with respect to the controls, particularly for the mesophilic aerobic population, lactic acid bacteria and *Pseudomonas* spp. While a diet supplemented with linseed oil has been demonstrated to affect the qualitative composition of the rumen microbiota, with a selective effect on specific population [52], no literature data are available on the effect of linseed on meat microbiota. Some authors proved the antimicrobial effect of selected fatty acids in vitro [53], while no results on the correlation between fatty acid composition and meat microbiota are actually available. Nevertheless, analysing the whole dataset, it appears that the compositional and chemical-physical changes determined on the meat characteristics by the linseed alone, and most of all by the linseed associated with vitamin E supplementation, improve the microbiological profile of the final products, extending their shelf-life. This is a crucial point, as minced-meat-derived products are highly exposed to microbial spoilage and can be stored for a very short time. On the contrary, including the essential oils within the burger’s formulation did not help to contain the microbial growth during time. In fact, in the tested conditions, the biopreservatives were ineffective in reducing the microbial load, and in several cases, they even stimulate the microbial growth. In accordance with Serio et al. (2010) [54], after a lag phase extension, sub-lethal concentrations of essential oils stimulate the growth of several bacteria. Moreover, some Specific Spoilage Organisms (SSOs), such as *Pseudomonas* spp., are particularly resistant to phenolic compounds, such as thymol [55], contained in *O. hirtum*, the EO applied in our study. Therefore, while the applied EO concentration is useful to reduce lipid oxidation, higher EO amounts could be necessary to contain the microbial growth.

Table 4 summarises the results of meat fatty acid composition. According to Albertì et al. (2013) [56], the levels of SFA, MUFA and PUFA in the meat and meat products could be ascribable to multifactorial effects. Moreover, other authors found different levels of FA in the meat of young bulls fed with flaxseed [57] compared to our results, or linseed [58] showing as breeds, production systems or slaughter weight can influence these parameters.

The levels of MUFA were affected by diet, but not by the essential oil treatment. This result agrees with previous studies [57,59] in which an increase in MUFA in meat of animals supplemented with unsaturated fatty acids were recorded. Even if the quantity of linseed was the same between the L and LE groups, the significant increase in MUFA and PUFA in the burgers of the LE group recorded here was mainly ascribable to the use of vitamin E. We observed the same trend for PUFA (*p* < 0.05), n-3 (*p* < 0.01) and CLA (*p* < 0.01) in the LE group compared to groups L and C. Other authors [58,59] confirmed that the diet enriched with linseed can affect the concentration of n-3 FA in meat as demonstrated also in our research between the L and LE groups compared to group C. The higher concentration of n-3 FA also contributed to increase the availability of linolenic acid in the muscle, resulting in an enhanced synthesis of its elongation and desaturation products, such as EPA (Eicosapentaenoic acid) and DHA (Docosahexaenoic acid). The higher value of I-Harris index is due to the higher EPA and DHA level in the muscle of animals that received a diet enriched with linseed. This is a very important aspect on the human health view because the higher level of healthy FA (I-Harris index and n-3) and lower SFA suggests that the hamburger from the L and LE groups are healthier compared to the C group [29]. We also registered a different concentration of these long-chain FA between groups L and LE, probably due to the use of vitamin E added to extruded linseed in the diet of the LE group compared to group L. The action of vitamin E in the rumen is not well known, but seems to influence the activity of Butyrivibrio Fibrisolvens, modifying the rumen PUFA biohydrogenation with a consequent increase in fatty acids, such as CLA, and the same PUFA [60,61].

The burgers from groups L and LE showed a higher CLA concentration compared to C group. These results are coherent with those reported in other studies [40,62,63], where diets rich in linoleic acids were demonstrated to increase the levels of C18:1 t11 acid in meat.

## 5. Conclusions

This study demonstrated that the use of linseed and vitamin E in the diet of Marchigiana young bulls had an important effect on colour and lipid stability of the burgers. Furthermore, EOs seem to protect the burgers from lipid oxidation through the antioxidant effects during storage. On the other hand, the applied EO concentration was not effective in reducing microbial spoilage over time. Nevertheless, linseed and vitamin E supplementation had a positive effect on microbial loads and growth dynamics, containing microbial development over time. These results seem to suggest that, to optimise the minced meat for burger preparation from Marchigiana bulls, the use of vitamin E is recommended. Indeed, the use of vitamin E in high doses (2,1 gr/head/d) with about 1 kg of extruded linseed in the finishing diet of young Marchigiana bulls improves the profile of fatty acids with beneficial effects also on the consumer’s health.

## Figures and Tables

**Table 1:** Ingredients of the three experimental diets: C (control), L (linseed) and LE (linseed + vitamin E).

Dietary Ingredients (Kg)	C	L	LE
Dehydrated Alfa Hay	2.86	2.85	2.85
Straw	1.14	1.14	1.14
Corn meal	4.57	4.56	4.56
Extruded linseed	0.00	0.97	0.97
Beans	1.14	0.46	0.46
Cereal bran	2.29	2.28	2.28
Hydrogenate fat	0.29	0.00	0.00
Sodium chloridae	0.06	0.06	0.06
Sodium bicarbonate	0.11	0.11	0.11
Vitamin E	0.00	0.00	0.02
Chemical composition (% DM)			
Dry matter	87.52	87.79	87.79
Crude fiber	6.82	6.86	7.91
Crude protein	13.87	13.91	13.91
Ether Extract	6.84	6.84	7.06
Ash	2.18	2.15	2.25
Fatty Acids Composition (% total fatty acids)			
SFA	51.16	14.84	16.72
MUFA	15.75	19.18	18.76
PUFA	33.09	65.98	64.52

**Table 2:** Effect of three experimental diets on meat quality chemical composition at time point 0.

	Diet				
	C	L	LE	*p*-Value	SEM
Moisture (%)	74.79	75.43	75.45	0.72	9.33
Protein (%)	21.70	21.05	21.02	0.66	3.32
Fat (%)	2.29	2.36	2.35	0.98	0.24
Ash (%)	1.22	1.16	1.18	0.68	0.15

C = Control; L = Linseed; LE = Linseed plus vitamin E; SEM = Standard error of the mean.

**Table 3:** Effects of diets (C, L and LE) and essential oils (O and WO) on Marchigiana beef burgers’ colour parameters (L, a*, b), pH, FRAP and vitamin E content.

	Diet			EO					*p*-Value			
	C	L	LE	O	WO	Diet	EO	Time	D*EO	D*T	EO*T	SEM
pH	5.70 ^A^	5.60 ^C^	5.67 ^B^	5.47 ^b^	5.65 ^a^	<0.01	0.03	<0.01	0.67	0.54	0.55	0.09
TBARS	0.47 ^ab^	0.60 ^a^	0.39 ^b^	0.43 ^B^	0.55 ^A^	0.05	<0.01	<0.01	0.41	<0.01	<0.01	0.01
Vitamin E mg/kg	0.64 ^c^	0.81 ^b^	1.38 ^a^	1.12	1.11	0.01	0.56	0.74	0.13	0.78	0.54	0.05
FRAP µmolFe/g	0.56 ^b^	0.55 ^b^	0.74 ^a^	0.67 ^b^	0.52 ^a^	0.03	0.05	0.51	0.24	0.56	0.38	1.02
L	43.85 ^B^	45.29 ^A^	43.40 ^B^	44.68 ^A^	43.68 ^B^	<0.01	0.77	<0.01	0.18	<0.01	0.12	1.05
a	19.39 ^B^	17.83 ^C^	20.68 ^A^	19.27	19.34	<0.01	0.92	<0.01	0.38	<0.01	0.57	1.18
b	12.65 ^A^	11.63 ^B^	12.90 ^A^	12.36	12.43	<0.01	0.70	<0.01	0.64	<0.01	0.88	1.21

C = Control; L = Linseed; LE = Linseed plus vitamin E; O = with essential oil; WO = without essential oil. D*EO = Diet*Essential Oils; D*T = Diet*Time; EO*T = Essential Oils*Time. Different letters on the same row indicate significant differences within diet or EO (^a–c^: *p* < 0.05; ^A–C^: *p* < 0.01). SEM = Standard error of the mean.

**Table 4:** Effects of experimental diets (C, L and LE) and treatment (EO) with (O) and without essential oils (WO) on health indices and pro-oxidant fatty acids on Marchigiana beef meat on days 0 and 12 of storage.

	Diet			EO		*p*-Value						
	C	L	LE	O	WO	Diet	EO	Time	D*EO	D*T	EO*T	SEM
SFA	956.99 ^a^	721.53 ^ab^	679.51 ^b^	766.78	705.24	0.05	0.06	0.27	0.15	0.58	0.60	1.06
MUFA	595.53 ^b^	642.10 ^ab^	699.22 ^a^	681.75	609.48	0.05	0.17	0.14	0.16	0.45	0.98	0.90
PUFA	203.22 ^b^	226.48 ^ab^	253.63 ^a^	221.31	234.25	0.05	0.40	0.42	0.90	0.33	0.45	1.26
n-3 PUFA	31.70 ^C^	49.43 ^B^	68.40 ^A^	52.16	47.52	0.01	0.15	0.14	0.56	0.20	0.68	0.14
n-6 PUFA	121.88	125.00	121.54	122.89	122.72	0.68	0.96	0.68	0.57	0.06	0.68	1.13
CLA	5.79 ^C^	11.22 ^B^	15.60 ^A^	11.90	10.84	0.01	0.06	0.58	0.55	0.06	0.27	0.94
I-HARRIS	7.25 ^b^	8.85 ^a^	8.21 ^a^	7.80	8.40	0.05	0.42	0.10	0.64	0.42	0.27	0.03

Fatty acids are expressed as mg/100 gr of samples. SFA = Saturated fatty acids. MUFA = Monounsaturated fatty acids. PUFA = Polyunsaturated fatty acids. N3-PUFA = Σ C18:2 t11, c15 + C18:2 c9, c15 + C18:3 c9, c12, c15 + C:22:5 c7, c10, c13, c16, c19 + EPA + DHA. N6-PUFA = Σ C18:2t9, t12 + C18:2 c9, t12 + C18:2 t9, c12 + C18:2 c9, c12 + CLA t10, c12 + C20:2 c11, c14 + C20:3 c8, c11, c14 + C20:4 c5, c8, c11, c14. CLA = conjugated linoleic acid. I-Harris = (EPA + DHA). Different letters in the same row indicate significant differences (^a,b^: *p* < 0.05; ^A–C^: *p* < 0.01). SEM = Standard error of the mean. C = Control; L = Linseed; LE = Linseed plus vitamin E; O = with essential oil; WO = without essential oil. D*EO = Diet*Essential Oils; D*T = Diet*Time; EO*T = Essential Oils*Time.

## Data Availability

The data supporting the findings of this study are available within the article.
